# Primary Closure of A Sternal Cleft in A Neonate

**Published:** 2016-09

**Authors:** Shaal Ramdial, Desigan Pillay, Anil Madaree

**Affiliations:** Department of Plastic and Reconstructive Surgery, Nelson R Mandela School of Medicine, University of KwaZulu-Natal, Durban, South Africa

**Keywords:** Sternal cleft, Bifid sternum, Chest wall deformity, Chest wall abnormality

## Abstract

A three day old neonate was referred to our department with a problem of a sternal cleft. Sternal clefts are often associated with a myriad of other abnormalities ranging from mild to severe. We present our experience with such a problem, and review the current literature concerning it.

## INTRODUCTION

The sternum is of mesodermal origin and development begins in the sixth week of gestation. A pair of mesenchymal bars develops ventrolaterally and, during the sixth to the ninth week of gestation, elongate and fuse medially to form a series of sternal bars. This occurs in a cranio-caudal direction, with subsequent chondrification – giving rise to the sternal body. Fusion beyond the last rib gives rise to the xiphoid process.^1^

The manubrium, by contrast, develops from three mesenchymal primordia – a central presternal mass and bilateral suprasternal masses. The presternal mass fuses with the cranial portion of the sternal bars, while the suprasternal masses go on to become the sternoclavicular joints. Ossification of the sternum also occurs in a craniocaudal direction. Ossification begins in the fifth month of gestation, however the xiphoid process usually only begins this process by the age of three. In light of the above, it would seem logical to assume that sternal clefting would occur in the inferior sternum; however the more common presentation is a cleft of the superior sternum. As such, it is assumed that superior sternal clefting is not simply a failure of fusion of the sternal bars, but rather a failure of formation of the manubrium itself.^2^

Sternal clefts account for less than one percent of all chest wall malformations and may be complete or partial. The partial defects are further divided into superior partial, inferior partial and sternal foramen defects. The superior partial defect is the most common, accounting for 67% of the patients. The complete, inferior partial and sternal foramen defects occur in 19.5%, 11% and 2.5% respectively.^2^ Patients are often diagnosed in the neonatal period; however some may reach adulthood without seeking medical assistance. Prenatal diagnosis of a sternal cleft and any associated abnormalities is possible.^3^ The sternal cleft behaves as a flail chest and patients sometimes become symptomatic (27%) – developing respiratory embarrassment with or without lower respiratory tract infections.^2^

Sternal clefts may be isolated (27%) or associated with other abnormalities (73%). With this in mind, a thorough examination should be performed prior to surgery. The most common associated conditions include cardiac defects (22.1%), aortic malformations (9.3%), Cantrell’s pentalogy (7%), PHACES syndrome (5.8%) and haemangiomas (cutaneous and/or visceral) (4.7%).^4^ Cantrell’s pentalogy is a syndrome which includes omphalocoele, anterior diaphragmatic hernia, ectopia cordis, cardiac abnormalities and sternal cleft. PHACES syndrome includes posterior fossa malformations, haemangiomas, arterial anomalies, cardiac defects, eye abnormalities and sternal cleft. We present primary closure of a sternal cleft in a neonate.^4^

## CASE REPORT

**Fig. 1:**
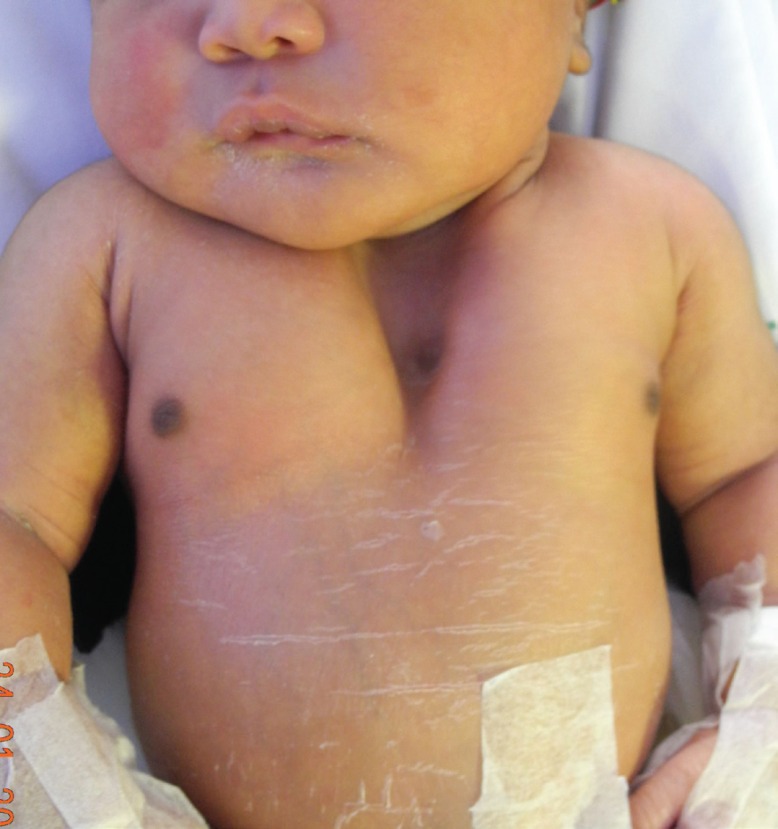
Pre-operative anterior chest wall appearance

A female neonate was referred from her district hospital to our quaternary facility soon after her birth, for a chest wall defect. She was admitted to the neonatal intensive care unit (NICU) where a diagnosis of a sternal cleft was made. At birth, the baby was 4.3kg and was delivered at term via a caesarean section for foetal distress. The baby’s Apgar scores were 8 and 9 at one and five minutes respectively, and she did not have any signs of respiratory distress. Clinical examination revealed a flat, cutaneous haemangioma overlying the sternal cleft. Examination of the chest revealed a cleft of the upper sternum (Figure 1). There was paradoxical recession of the cleft area on inspection. No other abnormalities were noted. Preliminary investigations included a cranial ultrasound which was normal and an echocardiogram that revealed a small pericardial effusion.

**Fig. 2:**
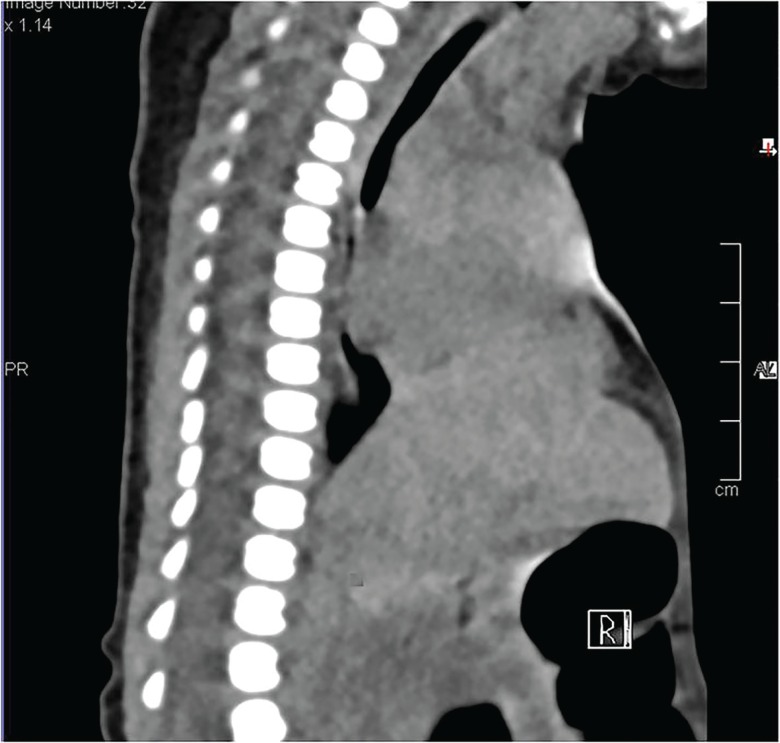
CT scan of chest – Sagittal view. Note the superior defect of the sternum

**Fig. 3:**
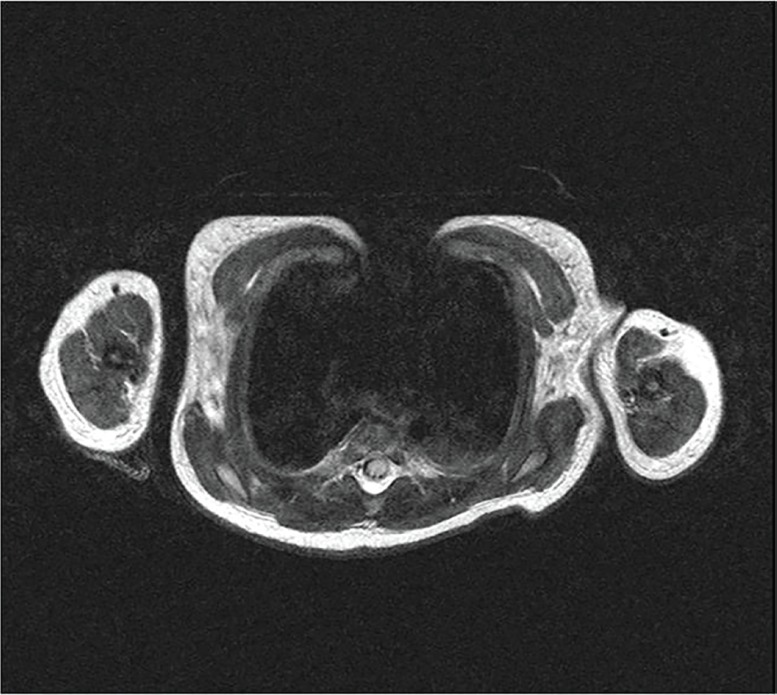
MRI scan of chest – Axial view. Note the anterior chest wall defect

**Fig. 4:**
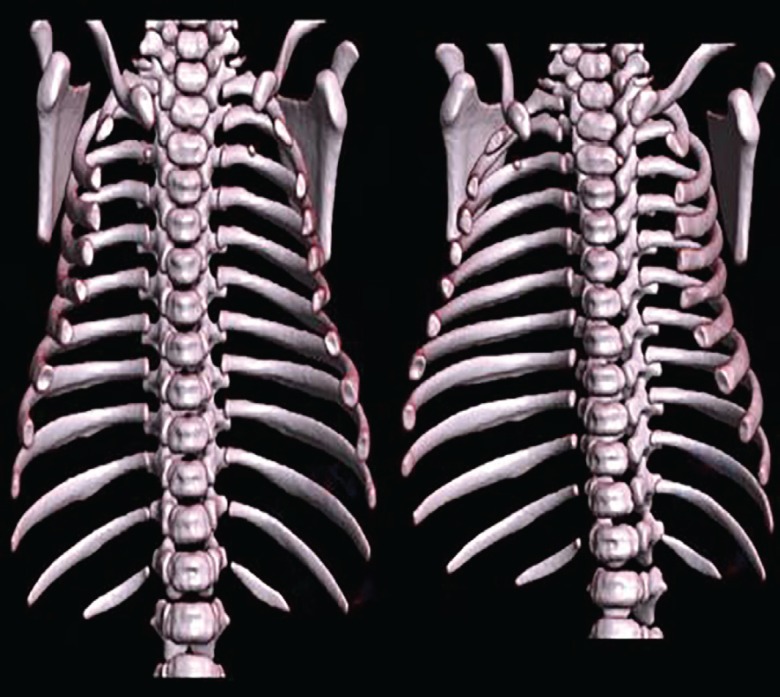
Three dimensional reconstruction of CT scan of chest

The mother was a healthy 34-year-old primigravida who diligently attended her local hospital’s antenatal programme. Rhesus factor, Wassermann reaction and HIV serology were all negative. There was no history of recreational or therapeutic drug use during the pregnancy. A full blood count from the neonate revealed a haemoglobin level of 17.7 g/dl, with normal platelet and white cell counts. Urea and electrolytes, liver function tests, calcium, phosphate and magnesium were all normal. Our team of plastic surgeons was consulted by the neonatal unit when the baby was 3 days old. At the time the baby was comfortable on room air, and a “wait and see” approach was adopted. CT and MRI scans were performed by day 7, revealing a defect in the superior portion of the sternum, with an overlying contour abnormality of the anterior thoracic wall (Figures 2-4).

**Fig. 5:**
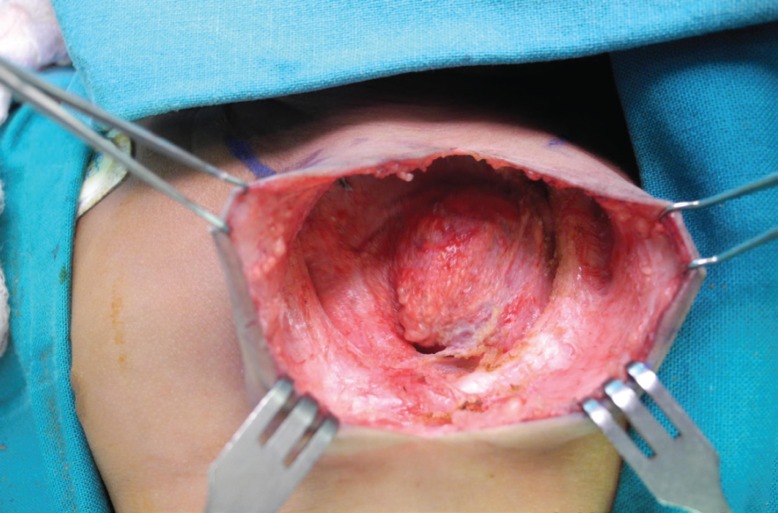
Intra-operative view revealing the U-shaped sternal defect

The baby gradually developed respiratory distress over the next week and, by day 18, was oxygen dependant. At this point, a multidisciplinary team (plastic surgeons, cardiothoracic surgeons, neonatologists and paediatric anaesthetists) was assembled to discuss a management plan. After extensive counseling, the mother consented to surgery. Consent was taken for a chest wall reconstruction with or without calvarial or rib bone grafts. Surgery was performed on day 23 of life. A midline thoracocervical incision was performed, which included the excision of the cutaneous haemangioma. Skin flaps were elevated off the pericardium. An iatrogenic pericardial tear was promptly repaired with a running polydioxanone 4/0 suture, after drainage of the pericardial effusion. After adequate exposure, a U-shaped superior sternal defect was noted (Figure 5). 

**Fig. 6:**
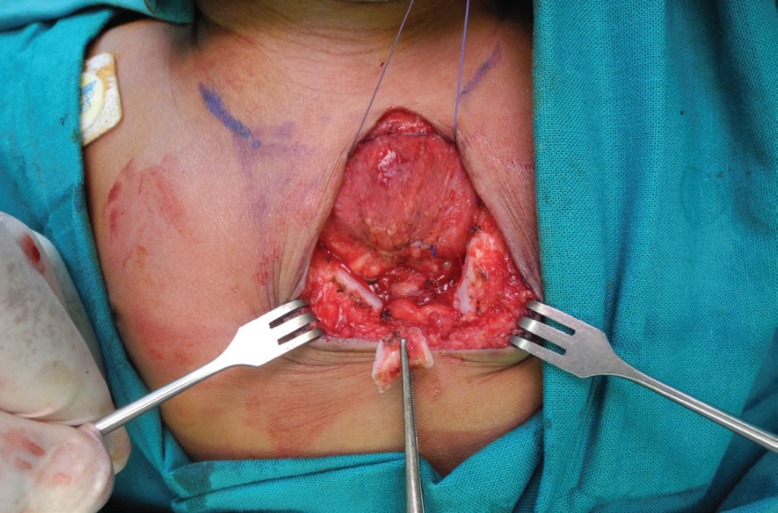
Intra-operative view showing V-shaped excision of lower sternum

**Fig. 7:**
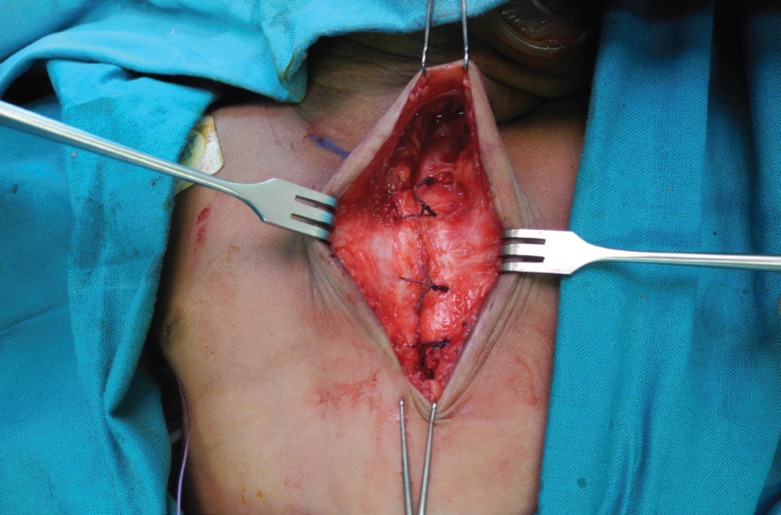
Intra-operative view displaying the primary closure of the sternal defect

A V-shaped wedge excision of the lower sternum was performed to allow for primary closure of the sternal defect (Figure 6). Four polyglactin 2/0 sutures were placed in the intercostal spaces. These sutures were pulled together but not tied. Heart rate, blood pressure, pulse pressure, oxygen saturation and airway pressures were monitored for five minutes. After a short period of haemodynamic and respiratory compromise, the aforementioned parameters returned to normal. The polyglactin sutures were then tied (Figure 7). The pectoralis major muscles were sutured to each other over the sternal repair with polyglactin 4/0 sutures. Thereafter, both sternocleidomastoid muscles were sutured to the superior portion of the reconstructed sternum, also with polyglactin 4/0 sutures. Interrupted poliglecaprone 4/0 sutures were placed in the dermis, and the same suture was used as a running subcuticular suture. The chest wall skin was splinted with an adhesive tape.

The baby was transferred back to the NICU intubated. Problems with endotracheal tube placement on day 0 after surgery resulted in intermittent drops in oxygen saturation. This ultimately led to prolonged assisted ventilation, and a lower respiratory tract infection. The infection was successfully treated, and the baby was extubated on day 7 after surgery. A wound inspection on this day revealed an intact suture line. By day 11 post-surgery, the baby was off oxygen and was transferred to the general paediatric ward. On day 15 post-surgery, the baby developed respiratory distress, requiring oxygen via nasal prongs. Chest x-ray revealed right perihilar consolidation and left upper lobe consolidation. The baby was started on intravenous clarithromycin and a combination of hypertonic saline and fenoterol nebulisation. Chest physiotherapy was commenced, and the baby was transferred to the paediatric high care unit.

The baby developed stridor and worsening respiratory distress on day 20 post-surgery. A diagnosis of laryngomalacia was entertained and the baby was transferred to the paediatric intensive care unit for treatment of the stridor with adrenaline nebulisation and for monitoring. By day 22 post-surgery, the baby’s condition had improved, and she was transferred back to the high care unit. A day later, the otorhinolaryngology team performed a flexible laryngoscopy. A generalised inflamed upper airway was noted, and no subglottic pathology was seen. A diagnosis of laryngomalacia was made, however the baby was not for any further surgical intervention.

**Fig. 8:**
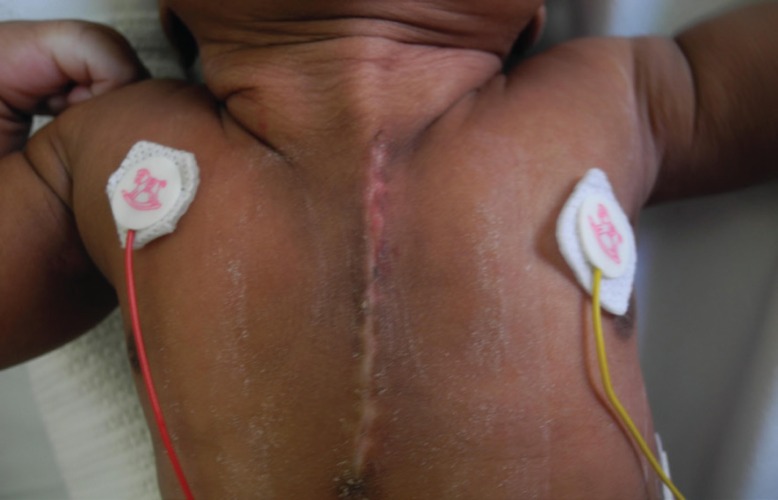
One month post-operative anterior chest wall appearance

**Fig. 9:**
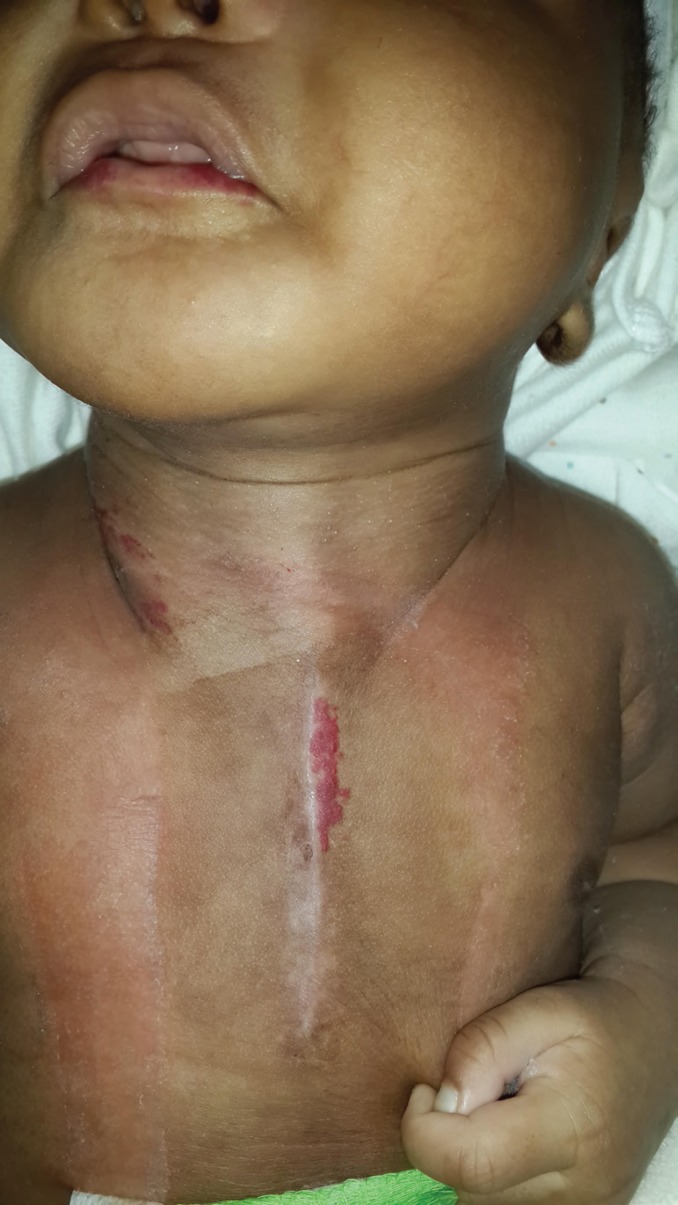
Two month post-operative anterior chest wall appearance

By day 27 the patient’s condition had stabilised and no further episodes of respiratory compromise were noted. The baby was discharged to her district level hospital for completion of her clarithromycin regimen. No sternal or cutaneous dehiscence was observed on discharge (Figure 8). The baby was reviewed a month later in our clinic. The sternal wound had completely healed and there was no bony defect palpable. New haemangiomas were seen on the neck, trunk and lower lip (Figure 9). The baby was otherwise healthy and growing well. Informed consent was taken from the mother of the child regarding the writing and subsequent publication of this article (including figures).

## DISCUSSION

The goals of sternal cleft repair are (i) Restore bony protection of the mediastinal structures, (ii) Prevent paradoxical movement of the chest wall and (iii) Address aesthetic concerns.^5^ Surgical closure of the sternal cleft is best performed in the neonatal period as this offers the surgeon the greatest chest wall compliance. Surgery during this window allows the cleft to be closed primarily, either alone or with soft and hard tissue manipulation (periosteal flaps,^6^ chondrotomies,^7^^,^^8^ cartilage resection). Primary closure accounted for 78.5% of the documented closure techniques in the literature. Should primary closure not be possible, other techniques will need to be employed. These include bone grafts and prosthetic closures. Alloplastic materials should be avoided though, as their use (and subsequent tissue reactions) has been associated with bradycardia, hypotension and infection. Bone graft may be harvested from rib, iliac crest, calvaria and tibia.^5^^,^^9^

Close cooperation with the anesthetic and neonatology teams is essential. Rapid intra-operative and sustained post-operative physiological changes occur which warrant intensive care monitoring post-operatively.^10^ The paediatric anaesthetist should be prepared for these changes, as well as the risk of cardiac, lung, great vessel and nerve injuries. Cardiac bypass should be available should the need arise. Sternal clefts are rare chest wall anomalies. Primary closure within the neonatal period is recommended, as the increased chest wall compliance allows for this. Delays beyond this period, may force the surgeon to employ additional techniques to facilitate this closure. A multidisciplinary team is mandatory in managing a patient with a sternal cleft, and regular pre-, intra- and post-operative input from all team members should result in a positive outcome.

## CONFLICT OF INTEREST

The authors declare no conflict of interest.
